# Supplementary material for: Mesenchymal Stem Cell-Derived Extracellular Vesicles as Proposed Therapy in a Rat Model of Cerebral Small Vessel Disease
Source: Int J Mol Sci. 2022 Sep 23;23(19):11211. doi: 10.3390/ijms231911211 (PMC9569832; doi:10.3390/ijms231911211)
Supplement: Supplementary file 1 [file ijms-23-11211-s001.zip › ijms-1908185-supplementary.pdf]

International Journal of
Molecular Sciences

Supplementary Materials:

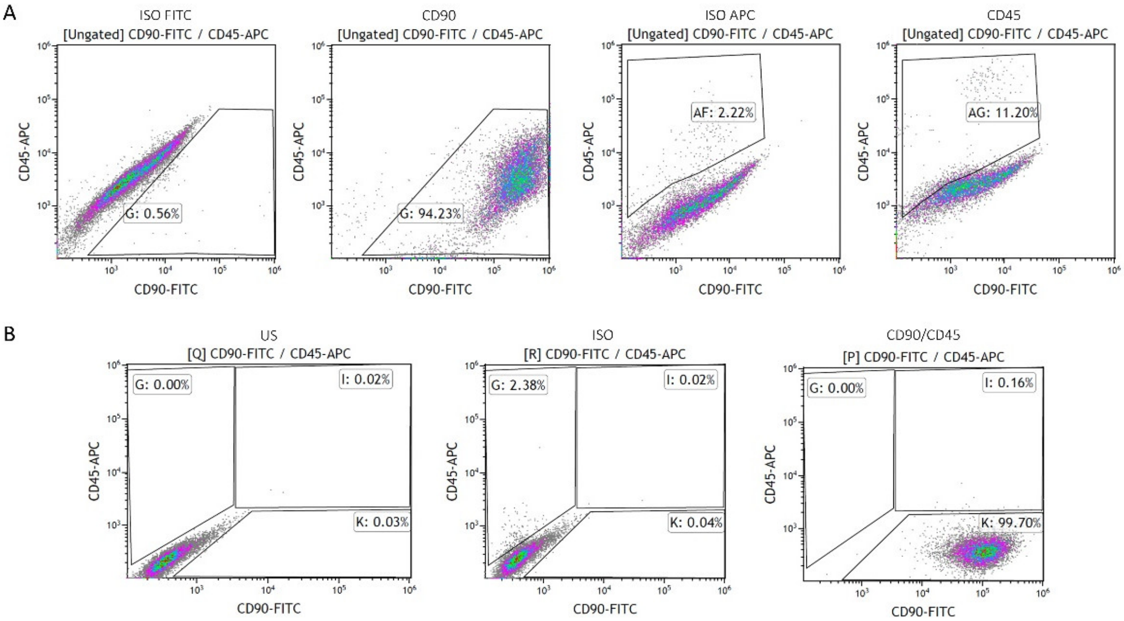

Figure S1. Mesenchymal stem cell (MSC) characterization following a hollow fiber-based bioreactor for cell culture. FACS analysis of CD90 and CD45 expression in P1 human adipose-derived MSCs. 94.23% of the isolated cells express the CD90 surface marker related to human MSCs. 11.2% of the isolated cells express the CD45 surface marker related to hematopoietic cells. Staining for nonspecific mouse immunoglobulin G (IgG) isotype fluorescence was used as a control (**A**). FACS analysis of human adipose-derived MSCs following cell growth in the bioreactor. 99.7% of the isolated cells express the CD90 surface marker related to human MSCs and are negative to CD45 surface marker related to hematopoietic cells. Incubation without antibodies was used as a negative control (US, unstained). Staining for nonspecific mouse IgG isotype fluorescence was used as a control (**B**).

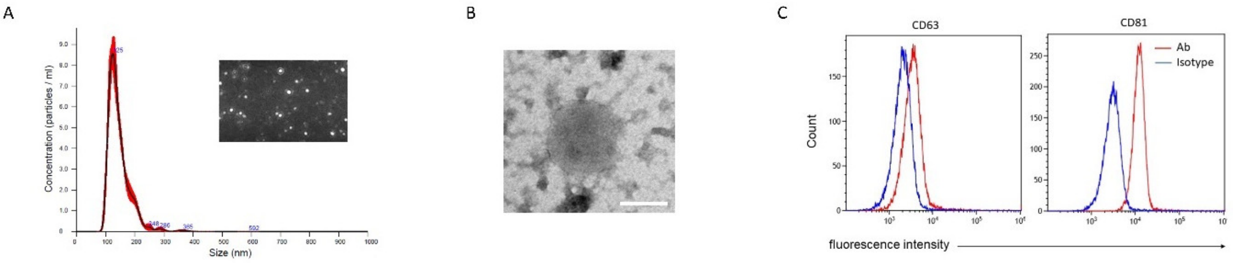

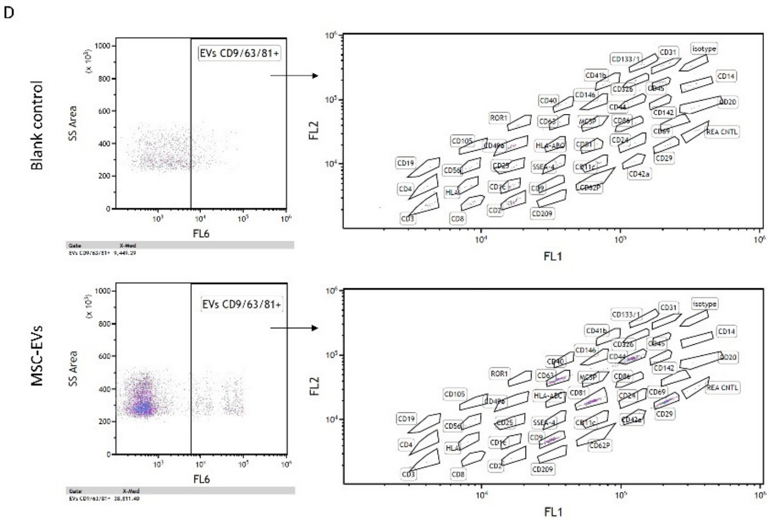

E

Initial cell count	20 Million
Scaffold (g)	2.8
Bioreactor working media volume (ml)	250
Process Duration (days)	26
Total media (L)	4
Net media for EV purification (L)	9.2
EVs /ml	$5.16x10^{11}$
EVs /ml media	$2.95x10^{9}$
Total EVs produced	$8.5x10^{12}$
Mean EV size (nm)	144.6±1.0
Final cell count (approximately)	1 Billion

Figure S2. Mesenchymal stem cell-derived extracellular vesicle (MSC-EV) characterization following a hollow fiber-based bioreactor for cell culture. Nanoparticle tracking analysis (NTA) distribution profiles of MSC-EVs, including size distribution plot and mean size (**A**). Representative transmission electron microscopy (TEM) image of MSC-EVs (**B**). Scale bar = 100 nm. FACS analysis of EV expression of surface molecules (**C**). EVs were loaded onto 4-μm-diameter aldehyde/sulfate latex beads and stained with CD63-APC or CD81-APC Abs (red lines) or negative control IgG1 Isotype Ab (blue line). MACSPlex analysis, applied to determine EV origin by surface protein composition (**D**). Top panel- blank control; Bottom panel- MSC-EVs. (**E**) A data table summarizes parameters of EV production from cells cultured in a bioreactor.

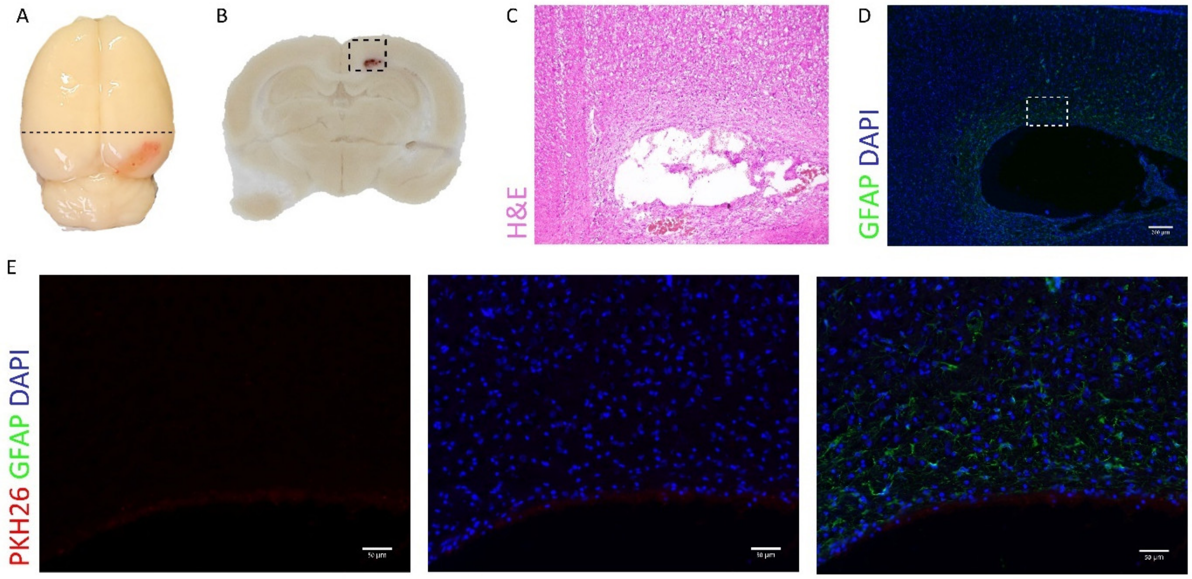

Figure S3. PKH26 fluorescent dye serves as a control for PKH26-labeled extracellular vesicle (EV) accumulation in lesion sites in brains of the SBH/y rat model of cerebral small vessel disease (CSVD). Representative images of brain derived from SBH/y DOCA rats following a single administration of PKH26 fluorescent dye (without EVs) (**A**). Dashed lines represent the coronal section presented in (**B**). 5x magnification of the injured area is presented using H&E (**C**) and GFAP immunostaining (**D**). Scale bar = 200 μm. A magnification of the inset in (**D**) is presented in (**E**). Scale bar = 50 μm.
